# The synthesis of *Paris* saponin VII mainly occurs in leaves and is promoted by light intensity

**DOI:** 10.3389/fpls.2023.1199215

**Published:** 2023-07-28

**Authors:** Feiyan Wen, Siyu Chen, Yue Wang, Qinghua Wu, Jie Yan, Jin Pei, Tao Zhou

**Affiliations:** ^1^ State Key Laboratory of Southwestern Chinese Medicine Resources, Chengdu University of Traditional Chinese Medicine, Chengdu, Sichuan, China; ^2^ College of Pharmacy, Chengdu University of Traditional Chinese Medicine, Chengdu, Sichuan, China

**Keywords:** *Paris* saponin VII, synthesis organ, leaf, light intensity, PPY

## Abstract

Unraveling the specific organs and tissues involved in saponin synthesis, as well as the light regulatory mechanisms, is crucial for improving the quality of artificially cultivated medicinal materials of *Paris* plants. *Paris* saponin VII (PS VII), a high-value active ingredient, is found in almost all organs of *Paris* plant species. In this study, we focused on *Paris polyphylla* var. *yunnanensis* (Franch.) Hand. - Mzt. (PPY) and found that PS VII synthesis predominantly occurs in leaves and is increased by high light intensity. This intriguing discovery has unveiled the potential for manipulating non-traditional medicinal organ leaves to improve the quality of medicinal organ rhizomes. The analysis of the impact of organ differences on saponin concentration in *P. polyphylla* var. *chinensis* (Franch.) Hara (PPC), *P. fargesii* Franch. (PF), and PPY revealed consistency among the three *Paris* species and was mainly dominated by PS VII. Notably, the leaves and stems exhibited much higher proportions of PS VII than other organs, accounting for 80–90% of the four main saponins. Among the three *Paris* species, PPY had the highest concentration of PS VII and was selected for subsequent experiments. Further investigations on saponin subcellular localization, temporal variation, and stem wound fluid composition demonstrated that PS VII is synthesized in mesophyll cells, released into the intercellular space through exocytosis, and then transported to the rhizome via vascular tissue. These findings confirm the significant role of leaves in PS VII synthesis. Additionally, a ^13^C-glucose feeding to trace PS VII biosynthesis revealed that only PS VII in the leaves exhibited incorporation of the labeled carbon, despite conducting ^13^C-glucose feeding in leaves, stems, rhizomes, and roots. Thus, the leaves are indeed the primary organ for PS VII synthesis in PPY. Furthermore, compared with plants under 100 μmol m^−2^ s^−1^, plants under 400 μmol m^−2^ s^−1^ exhibited a higher PS VII concentration, particularly in the upper epidermal cells of the leaves. We propose that high light intensity promotes PS VII synthesis in leaves through three mechanisms: (1) increased availability of substrates for saponin synthesis; (2) protection of leaves from high light damage through enhanced saponin synthesis; and (3) enhanced compartmentalization of saponins within the leaves, which in turn feedback regulates saponin synthesis.

## Introduction

*Paris*, a genus belonging to the Melanthiaceae family, is predominantly distributed in Asia with a partial presence in Europe. Throughout Asia, the wild resources of *Paris* are in a critically endangered state due to overexploitation and habitat degradation. Recognizing this threat, the International Union for Conservation of Nature (IUCN) has designated *Paris* as ‘vulnerable’ on its Red List, highlighting the decline in wild populations. In China, the entire genus *Paris*, with the exception of *Paris verticillata* M.-Bieb., is listed under special state key protection for wild plants. At present, the artificial cultivation industry of *Paris* species has achieved a certain scale. The main cultivated species are *Paris polyphylla* var. *yunnanensis* (Franch.) Hand. - Mzt. (PPY) and *Paris polyphylla* var. *chinensis* (Franch.) Hara (PPC), which are listed in the Chinese Pharmacopoeia. *Paris* species all contain Paris saponins (PS), which are isoprene compounds that exhibit a diverse range of pharmacological activities, including antioxidant, anticancer, anti-inflammatory, antifungal, and hypolipidemic functions (Qin et al., 2018; Ding et al., 2021). However, despite cultivation periods lasting nearly 8 years, the saponin concentration in most cultivated medicinal materials of *Paris* fails to meet the minimum standards required for medicinal use (Wu et al., 2017). This discrepancy raises concerns about the environmental adaptability of *Paris* plant growth and the synthetic biology of saponin, particularly the lack of clarity regarding the synthesis organelle. These factors serve as key limitations in achieving high-quality medicinal materials of *Paris*.

The principal saponins identified in *Paris* species comprise *Paris* saponin VII, H, VI, II, I, Dioscin, and Gracillin (Zhou et al., 2021). In the current version of National Pharmacopoeia Commission, (2020), PS VII, PS II, and PS I are designated as quality control components for medicinal materials of *Paris* (*Paridis Rhizoma*). In addition, PS H is recognized as an important hemostatic active ingredient (Bi et al., 2021). Typically, the *Paris* rhizome with the highest saponin concentration and biomass accumulation is used as medicinal material. However, studies have revealed the presence of PSs in various organs of PPY, including the root, stem, leaf, flower, fruit, and seeds. Analysis of saponin types in different organs has shown a consistent composition of PS VII in leaves and stems, which differs from that found in the rhizome. Moreover, the concentration of PS VII in the rhizome increases gradually during the initial years of growth (usually within the first 4 years) but remains lower than that in the leaves (Wen, unpublished data). Notably, young *Paris* plant leaves have the highest concentration of PS VII (Wen unpublished data). These findings provide evidence supporting the significant role of leaves in the synthesis of PS VII.

Leaves have been speculated to be a major organ for the synthesis of steroidal saponins in medicinal plants. For instance, the subcellular localization of ginsenoside Rb_1_ in *Panax ginseng* has been identified in chloroplasts, the cytoplasm of parenchymal cells, and the vascular phloem cells of leaves (Yokota et al., 2011). In *Dioscorea* species, including *Paris* species, Diosgenin saponins play a pivotal role. In *Dioscorea zingiberensis*, the distribution of diosgenin saponins is predominantly observed in multiple plant tissues. These include the epidermis of the leaves, the palisade tissue, spongy tissue, phloem in the vascular tissues of the stem, and the ground tissue within the rhizomes. (Li et al., 2022). By analyzing spatial and temporal variations, immunohistochemical localization, and transcriptome data, it has been revealed that diosgenin is first synthesized in the leaves of *Dioscorea zingiberensis*, converted into dioscin, and subsequently transported to the rhizome for storage (Li et al., 2022). Similarly, in PPY, transcriptome analysis has indicated that genes responsible for regulating PS synthesis are specifically expressed in the leaves where they are significantly more highly expressed than in the rhizome (Gao et al., 2020). Based on these findings, we hypothesize that in PPY, the leaves, rather than the rhizome, serve as the primary organ for the synthesis of PS.

Despite extensive research on the pharmacological activities of saponins in disease treatment, our understanding of their biological functions, particularly their synthesis, distribution, and accumulation in response to biotic and abiotic stress, remains limited. Unlike mobile organisms, plants growing in soil have developed mechanisms to protect themselves from external damage, often utilizing specialized metabolites (Liu et al., 2019a; Hunziker et al., 2021). Optimization of chemical defenses is one strategy employed by plants to defend against herbivores (Gershenzon and Ullah, 2022), and saponins have been identified as a class of defense compounds that exhibit toxicity or deterrent effects on insects, molluscs, fungi, and microorganisms (Faizal and Geelen, 2013; Hussain et al., 2019; Ninkuu et al., 2021). The unique chemical structure of saponins, characterized by a hydrophobic terpenoid backbone with attached hydrophilic saccharide groups, contributes to those functions by altering cell membrane permeability, ultimately leading to cell damage (Stewart et al., 2018).

In addition to their role in response to biotic stress, saponin biosynthesis can be induced to mitigate abiotic stressors, such as light, temperature, and drought (Szakiel et al., 2011; de Costa et al., 2013; Rogowska and Szakiel, 2020). The natural habitat and cultivation areas of PPY primarily encompass Yunnan and the plateau of western Sichuan in China, with total solar radiation ranging from 5,000 to 5,850 MJ m^−2^ per year, which is approximately the annual average for China as a whole (5,900 MJ m^−2^). Interestingly, PPY grown in areas with lower light intensity, such as Sichuan and Guizhou in China, where total solar radiation ranges from 3,350 to 4,190 MJ m^−2^ per year, exhibits significantly lower saponin concentrations than PPY grown in Yunnan. Previous studies have shown that optimizing light quality and intensity can increase the total saponin concentration and rhizome biomass of PPY (Zhang et al., 2018). Additionally, LED light exposure has been found to increase the accumulation of triterpenoid saponin glycosides in *in vitro* culture of *Bacopa monnieri* when combined with precursor feeding (Watcharatanon et al., 2019), while saponins in *Centella asiatica* have been shown to increase resistance to UV oxidation damage (Müller et al., 2013).

Traditional Chinese medicine and crops have played a significant role in promoting human health and survival. However, our understanding of the growth habits and survival strategies of medicinal plants lags behind that of crops. This knowledge gap has resulted in outdated cultivation techniques, small-scale production, and an inconsistent quality of medicinal herbs. Although advancements in sequencing technologies and molecular biology have led to the identification of whole-genome sequences of over 100 medicinal plant species and the elucidation of synthetic pathways for important medicinal components, their impact on high-quality herbal medicine production has been limited. To address this issue, it is essential to gain insights into the tissue-specific and environmentally regulated mechanisms underlying the synthesis of medicinal components. Such understanding is a prerequisite for developing cultivation practices that achieve both high quality and high yield in medicinal plants. To bridge this knowledge gap, a series of experiments were conducted using plant physiological and biochemical analysis methods. These included variability analysis at the tissue level, spatial and temporal specificity analysis, isotopic ^13^C labeling, and transmission electron microscopy observation. The aim was to clarify the main organs responsible for the synthesis of PS and how their synthesis is regulated by light intensity. Specifically, two hypotheses were tested: (1) the leaf is the main organ for the synthesis of PS, and (2), high light intensity increases the synthesis of PS in leaves.

## Materials and methods

### Plant materials

The study employed 5-year-old plants of three *Paris* species: *P. polyphylla* var. *chinensis* (PPC), *P. fargesii* (PF), and *P. polyphylla* var. *yunnanensis* (PPY). These plants were collected from different locations in China: PPC from Sichuan Pengzhou (103°44′E, 31°13′N), PF from Sichuan Dujiangyan (103°33′E, 30°55′N), and PPY from Yunnan Wenshan (104°11′E, 23°04′N). Analysis of saponins in the leaves, stems, rhizomes, and roots of these plants was conducted to evaluate their similarities and differences. Additional 5-year old *P. polyphylla* var. *yunnanensis*(PPY) plants were collected for a leaf saponin transport experiment and the sampling of stem wound fluid. To avoid interference from saponin accumulation in perennial rhizomes, 2-year-old seedlings were used for the ^13^C-glucose (^13^C_6_H_12_O_6_) feeding and light intensity experiments. The weight of the seminal root of the seedlings was controlled at approximately 2 ± 0.2 g. The seedlings were initially harvested from Wenshan, Yunnan during winter and then cultivated in a climate chamber. The following year, seedlings of the same height were selected for the experiments after the plants had achieved stable growth in height.

### Experimental design and plant management

The experiment was conducted using a randomized block design with three replicates in a greenhouse environment. The greenhouse maintained a relative humidity of 75% and a day/night temperature of 20°C/10°C, and a photoperiod of 12 h day/12 h night during the plant growth period. PPY has the characteristics of a typical heliophyte herb; according to the daily sunshine hours during the growing period in Yunnan, a photoperiod of 12 h day/12 h night was employed. The irradiance was provided by light-emitting diode (LED) lamps. Our previous study found that 2-year-old PPY seedlings can tolerate a light intensity of up to 400 μmol m^−2^ s^−1^ for cultivation in the laboratory (data not shown), with higher photosynthesis and only slight photoinhibition. Thus, light intensities of 100 and 400 μmol m^−2^ s^−1^ were chosen as the low and high light intensity treatments, respectively. All seedlings were placed under 100 μmol m^−2^ s^−1^ before treatment. During the plant growth period, the distance gap between LED lamps and the plant canopy was 30 cm. Irradiance was measured routinely using a quantum sensor (Highpoint, Taiwan).

The 5-year-old PPY were individually placed in pots measuring 10×10×10 cm. Six PPY were used for the leaf saponin transport experiment and three for the stem wound fluid sampling. The 2-year-old seedlings were placed in seedling disc of 25 holes; 100 seedlings were used under each light intensity (100 and 400 μmol m^−2^ s^−1^) in follow-up experiments. All seedlings received regular watering and nutrient supply according to their respective treatments.

**Figure 1:**
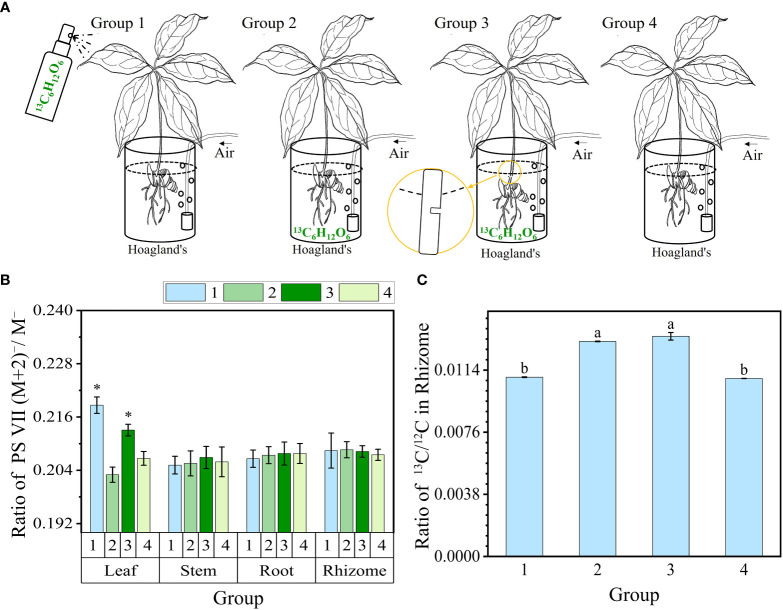
^13^C-glucose (^13^C_6_H_12_O_6_) feeding track PS VII biosynthesis via plant organs. **(A)** Schematic diagram illustrating glucose feeding in different organs. Groups 1–3 were fed in the leaf, rhizome, and stem vascular bundle, and group 4 was not fed. **(B)** The ratio of PS VII (M+2)^−^/M^−^ in organs. **(C)** The ratio of ^13^C/^12^C isotopes in rhizomes of four groups. PS, *Paris* saponins. Each column represents the mean (± SE) of three replicates. * indicates a significant difference (t-test at *p ≤ 0.01*). Data with different letters are significantly different between four groups (*p≤0.05*).

The experiment involving ^13^C-glucose (^13^C_6_H_12_O_6_) feeding track PS VII biosynthesis via plant organs was set to four groups as follows (**Figure 1A**). In group 1, the leaves were sprayed with 0.2%^13^C-glucose solution at a volume of 1 ml per leaf, administered once every 2 days (Yuan et al., 2020). In groups 2 and 3, the roots of the seedlings were fed with 0.2%^13^C-glucose solution. Additionally, in Group 3, the stem of the seedlings was partially severed near the rhizome end to facilitate feeding through the stem’s vascular bundle. Group 4 served as the control group without any ^13^C-glucose treatment. The experimental treatment time lasted for 10 days. In each group, three 2-year-old seedlings were subjected to a light intensity of 100 μmol m^−2^ s^−1^. All groups were cultivated hydroponically using Hoagland’s nutrient solution; the culture medium was changed every 2 days and the solution was ventilated with an air pump (Zhou et al., 2016).

### Plant sampling

**Figure 2:**
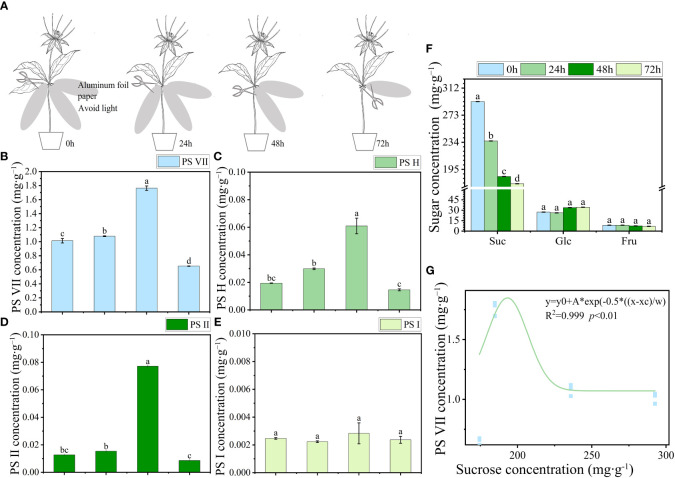
The transport characteristics of saponins and soluble sugar in PPY leaves. **(A)** Schematic diagram illustrating the use of aluminum foil for covering and sampling leaves. **(B–E)** Concentration of PS VII, PS H, PS II, and PS I, respectively. **(F)** Soluble sugar concentration in leaves. **(G)** Correlation between saponins and sucrose concentration. Each column represents the mean (± SE) of three replicates. PS, *Paris* saponins. Data with different letters are significantly different (*p ≤* 0.05).

**Figure 3:**
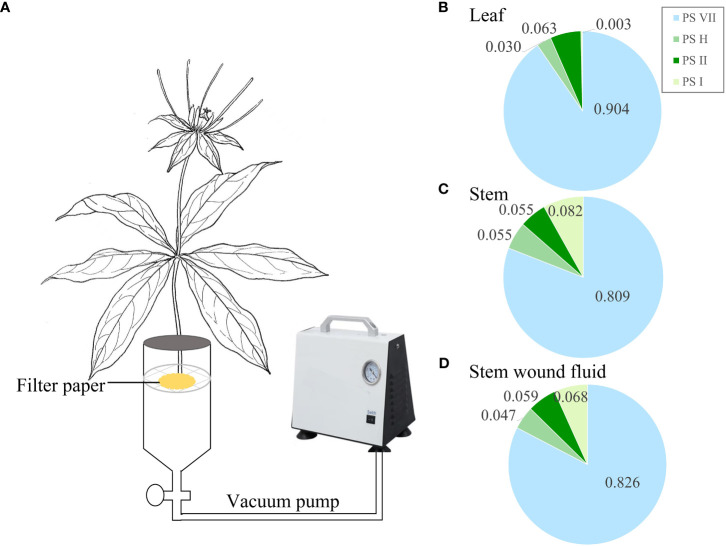
Saponin composition in stem wound fluid. **(A)** Schematic diagram illustrating the collection of stem wound fluid from wounded stem. **(B–D)** Proportion of the concentrations of the four main saponins in leaf, stem, and stem wound fluid, respectively. PS, *Paris* saponins. Each column represents the mean (± SE) of three replicates.

Four organ samples of the three *Paris* species were collected from origin for PS analysis. To investigate the PS flowing in the leaves, a specific procedure was followed for leaf sampling. Each seedling’s three leaves were individually wrapped in aluminum foil to avoid exposure to light (**Figure 2A**). Sampling was conducted daily at 5 pm, with a 24-h interval time series (0, 24, 48, and 72 h) followed. For the collection of stem wound fluid, the stem of the seedlings was severed near the rhizome end to obtain the fluid. A negative pressure pump with a pressure of 0.05 MPa was used to facilitate the outflow of the stem wound fluid within a few minutes. The fluid was then concentrated on 2 cm^2^ filter paper and immediately subjected to 75% ethanol extraction for the analysis of saponins (**Figure 3A**). Fresh PPY leaves were sampled and fixed for PS subcellular chemical localization using a transmission electron microscope (TEM).

At the conclusion of the ^13^C-glucose experiment, which lasted 10 days, the leaves, stems, rhizomes, and roots of each group were collected individually. These collected plant organs were thoroughly washed to remove any surface impurities. Subsequently, quantitative analysis of PS VII and the ratio of ^13^C/^12^C in different plant organs was performed. In the following light intensity processing experiment, the light intensities of two groups were set as 100 μmol m^−2^ s^−1^ and 400 μmol m^−2^ s^−1^. Throughout the 1-week treatment period, various photosynthetic parameters and chlorophyll fluorescence were measured. Then, the seedlings from the two groups were sampled to determine chlorophyll concentration. To examine cell morphology and chloroplast arrangement, the leaves were fixed with formalin-aceto-alcohol (FAA) solution, stained with red and solid green, and then sliced for observation under a general microscope. The distribution of saponins in the upper and lower epidermis was observed using chemical chromogenic techniques via TEM. At the same time, the upper and lower epidermis of the leaves were manually separated using pointed tweezers (approximately 2 mg of dried weight). Sections of leaves of considerable weight without major vascular tissue were removed by scissors. Subsequently, whole leaves without petioles, stems, rhizomes, and roots were collected separately. All samples for quantification were first incubated at 105°C for 30 mins and then dried at 60°C until a constant weight was reached.

### Measurement of *Paris* saponin VII, H, II, and I

The dried samples were ground to a fine powder using a steel ball mill (Tissuelyser-48, Jingxin, China). Approximately 30 mg of powder of each sample was subjected to ultrasonic and extracted in 2 ml of 75% v/v ethyl alcohol water at 25°C for 20 min twice, and three parallel samples were prepared. The extract solution was filtered, merged and dried with nitrogen, and then dissolved to a volume of 1 ml with chromatographic methanol. The extraction and preparation process for the filter-paper-adsorbed stem wound fluid was the same as with other organs. The preparation method for the upper and lower epidermis tissue was the same as above, except the dose was approximately 2 mg and the final volume was 200 μl. Before injection, a 0.22-μm organic phase filter (Jinteng, China) was used for filtration. Saponins were identified through comparison with retention times and coelution of authentic standard solutions. The main saponins were identified through qualitative analysis of the filter-paper-adsorbed stem wound fluid and other organs. The proportions of PS VII, PS H, PS II, and PS I in the sum of the four main saponins were calculated to compare the similarity of the saponin types and the proportion difference. In other experiments, quantitative concentrations of PS VII, PS H, PS II, and PS I were determined.

The determination of four saponins was carried out using a UFLC-MS/MS system, equipped with two LC-30D pumps, an SIL-30AC autosampler, a CTO-20AC column oven (Simadzu, Japan), and Qtrap 5500 mass spectrometry coupled with an electrospray ionization (ESI) interface, controlled by Analyst 1.6.2 software for operation, data acquisition, and analysis (AB SCIEX, USA). Chromatographic separations were performed on a Waters Acquity UPLC BEH C_18_ column (100 mm × 2.1 mm, 1.7 μm) at 35°C. The mobile phase was composed of 0.01% ammonia water in water (solvent A) and acetonitrile (solvent B) with a gradient elution: 0–1.0 min, 10–90% B; 1–2.5 min, 90% B (Yang et al., 2017). The flow rate was 0.5 ml min^−1^. The volume of the injection was 0.1 μl.

The mass spectrometer was operated in negative mode. The operating parameters of the ion source were designed as follows: ion spray voltage, −4.50 kV; source temperature, 500°C; curtain gas, 20 psi; ion source gas1, 60 psi; ion source gas2, 60 psi; and declustering potential, 100V. The multiple reaction monitoring (MRM) transitions were employed for quantification. Ion pairs of 1029.5–737.3, 869.4–721.3, 1013.5–721.3, and 853.4–721.4 were selected for the quantification of VII, H, II, and I, with collision energies of −60, −42, −59, and −44, respectively.

### Determination of sucrose, glucose and fructose concentration

Sucrose, glucose, and fructose concentration were determined in leaves. Dried leaves were extracted in 80% v/v ethanol at 80°C for 10 min. Before loading to an HPLC system, the extracts were filtered through 0.22 μm water phase filters (Jinteng, China). The HPLC assays of sucrose, glucose, and fructose were run on a Waters Binary HPLC System (Waters 1525–2707, Milford, USA) equipped with a refractive index detector (2414, Waters, Milford, USA). The analytical conditions were as follows: column Agilent Hi-Plea Ca (8% crosslinked) 300 mm × 7.7 mm, 8 µm in diameter (Agilent Technologies, Inc., USA); column temperature of 85°C; mobile phase Milli-Q water; and a flow rate of 0.6 ml min^−1^ (Zhou et al., 2022). Data were collected and processed by the Waters chromatography station Data Apex. Sugars were identified through comparison with retention times and the coelution of authentic standard solutions.

### ^13^C-glucose (^13^C_6_H_12_O_6_) feeding track PS VII biosynthesis via plant organs

High-resolution liquid chromatography-mass spectrometry was applied to track ^13^C-PS VII biosynthesis via plant organs. LC-MS can analyze a variety of substances in complex samples, and high-resolution mass spectrometry has the advantage of resolving isotope peaks. The exoisotope-labeled compounds have the same molecular structure and similar molecular weights, physicochemical properties, and retention times as the unlabeled compounds. Therefore, in the detection of PS VII labeled with different amounts of ^13^C molecules, extraction of the target molecule ion by high-resolution mass spectrometry can be used to resolve isotope peaks and calculate the ratio of PS VII labeled with different amounts of ^13^C. Owing to the natural abundance of ^13^C, there will be a series of small peaks exiting in the position of the PS VII molecular ion peak (M^−^), mainly (M+1)^−^, (M+2)^−^, (M+3)^−^, (M+4)^−^, etc. These are the isotope ion peaks of PS VII, which are mainly caused by the presence of ^13^C. Owing to the constant ratio of ^13^C isotope in the natural state, the ratios of (M+1) ^−^/M^−^, (M+2) ^−^/M^−^, (M+3)^−^/M^−^, and (M+4)^−^/M^−^ are generally fixed. When compounds labeled with exogenous ^13^C were entered into the mass spectrum and detected, the abundance of total ^13^C increased and the ratio of series isotope ion peaks changed. Therefore, in this study, the plants were fed with ^13^C-glucose (^13^C_6_H_12_O_6_) and PS VII was synthesized with an exogenous ^13^C label; the ratio of the series isotope ion peak changes were used to track and infer the biosynthesis of saponins. Owing to its advantages of high sensitivity, simplicity, and stability, this isotope tracking method has been widely used in drug chemical synthesis and absorption, distribution, metabolism, and elimination *in vivo* (Lozac'h et al., 2018; Martin et al., 2020).

The liquid chromatographic separation conditions were as follows: samples were eluted with the mobile phases of 0.1% formic acid water (A) and acetonitrile (B) with a BEH C18 column (1.7 μm, 2.1 × 100 mm, Waters, Milford, MA, USA) maintained at 40°C. The optimized gradient elution had a flow rate of 0.3 ml min^−1^, and the optimized gradient procedure was designed as follows: started with 10% B and linearly increased to 30% B for 8 min, then linearly increased to 45% B from 8 min to 16 min, linearly increased to 50% B from 16 min to 24 min, and linearly increased to 70% B from 24 min to 30 min. Finally, the gradient was rapidly decreased to its initial condition for 0.1 min then maintained at 10% B for the next 2.9 min. Samples of a 5 μl volume were injected for analysis (Wang et al., 2021).

Mass spectrometry conditions were as follows: All MS and MS/MS data were acquired from a Thermo QExactive Plus (Thermo Fisher Scientific, San Jose, CA, USA), which is equipped with a heated electrospray ionization source (HESI) via negative ion mode. The parameters of the HESI source were set as follows: capillary voltage, 3.0 kV; capillary temperature, 350°C; sheath gas (N_2_), 35 arbitrary units; and auxiliary gas (N_2_), 15 arbitrary units. Through full scanning (operated from *m/z* 100–1500), saponins were identified, and the appropriate collision energy was given to obtain the clear fragmentation pattern of the plausible saponins. According to the fracture rule, the structures of saponins were predicted. Xcalibur 4.2 software (Thermo Fisher Scientific, USA) was used for data acquisition and processing. The raw data files of the control and sample groups and blanks were imported into Compound Discoverer™ 3.0 software (Thermo Scientific, Fremont, CA, USA) to identify saponins by accurately calculating the mass and fragments of saponins, and the maximum tolerance of mass error was set at 5 ppm.

A negative ion mass spectrum of PS VII (C_51_H_82_O_21_, 1030.5349) was detected and mainly ionized with COOH^-^ ions in formic acid water (**Figure S1**). To rule out the effect of ^13^C natural abundance, the ratio of both marked and unmarked ion currents was calculated in each organ of the four groups. The calculation method of the ratio is the abundance of the series isotope ion peaks with ^13^C and the PS VII molecular ion peak (M^−^), i.e., (M+1)^−^/M^−^, (M+2)^−^/M^−^, (M+3)^−^/M^−^, and (M+4)^−^/M^−^. M^−^ means the sum of the peak areas of ion current (M+COOH) ^−^ and M^−^, which were mainly ionized with COOH^−^ ions in formic acid water. The calculation formula is illustrated as follows:


$$\text{Ratio}={\left(\text{M}+2\right)}^{-}/{\text{M}}^{-}\text{PS}:{\left(\text{M+}2\right)}^{-}\text{include}{\left(\text{M}+2+\text{COOH}\right)}^{-}\text{and}{\left(\text{M+}2\right)}^{-};{\text{M}}^{-}\text{ include}{\left(\text{M}+\text{COOH}\right)}^{–}{\text{and M}}^{-}.$$

The ^13^C/^12^C ratio in rhizomes of four groups were analyzed by gas chromatography combustion isotope ratio mass spectrometry (GC/IRMS, Delta V Advantage, Thermo Fisher, Germany). Dried rhizome was grinded through a 200-mesh sieve, and 0.2 mg of powder sample was wrapped in a tin cup. Solid samples were placed into the combustion tube and converted into CO_2_, which was separated by GC and detected by IRMS. Isodat software (Thermo Fisher Scientific, Germany) was used for data acquisition and processing.

### Leaf tissue distribution of saponins by transmission electron microscopy

Leaf saponins for cytochemical localization were treated as following (Chen et al., 2019): leaf samples were cut into 1-mm^3^ fragments and fixed with 3% glutaraldehyde (prepared with 3% lead acetate in 0.1 M sodium cacodylate buffer at a pH of 7.4) at 4˚C. Samples were then washed four times for 30 min each time with 3% lead acetate, following which, the cuts were incubated with 1% osmic acid (prepared with 3% lead acetate in 0.1 M sodium cacodylate buffer at a pH of 7.4) overnight at 4˚C, then washed twice (each for 30 min) with 0.1 M sodium cacodylate buffer at a pH of 7.4. The fragments were then gradually dehydrated with a grade series of ethanol of 30%, 50%, 70%, 80%, 90%, 95%, and 100%. The 100% ethanol step was repeated three times. Next, samples were incubated with epoxy propane transition and embedded with Epon812. The fragments were sliced into semi-lamellar sections to enable the positioning of the leaf cross-sections under a light microscope. Then, the semi-lamellar of the leaves was cut into 60–90-nm long sections using an ultramicrotome UC7rt (LEICA, Germany). After samples were stained with uranyl acetate, the sections were subsequently examined and photographed using a transmission electron microscope JEM-1400-FLASH (JEOL, Japan). The control sample was subjected to the same procedures as described above, but the fixing step with lead acetate was excluded.

### Determination of chlorophyll concentration, fluorescence, and photosynthetic rate

The fresh leaf samples were ground in liquid nitrogen, and 0.1 g of powder was extracted in 10 ml of 80% v/v aqueous acetone at 25°C in the dark for 48 h. Chlorophyll a+b (Chl a+b) and Chlorophyll a/b (Chl a/b) were measured directly in the extract at 663, 645, and 470 nm using an enzyme-labeled instrument (SpectraMax iD3, Molecular Devices, USA) (Arnon, 1949; Porra et al., 1989; HK and Buschmann, 2001). The chlorophyll fluorescence parameters maximal photochemical efficiency (*Fv/Fm*) and non-photochemical quenching coefficient NPQ(Fm/Fm’-1) were measured using a MINI-PAM-II portable fluorometer (Walz, Effeltrich, Germany). Before the measurement, the leaves required dark adaptation for 30 min, and the photochemical light was set to 178 μmol m^−2^s^−1^. Maximum photochemical efficiency (*Fv/Fm*), apparent electron transfer rate (ETR), and other chlorophyll fluorescence parameters of PS II were determined according to the instructions. Three plants were measured for each treatment and the average value was obtained. The photosynthesis rate (Pn) and stomatal conductance (Cs) were measured using a Li-6400 photosynthesis system (Li-COR, Lincoln, NE, USA). The measurements were taken on a sunny day in a greenhouse between 10:00 h and 12:00 h. The photosynthetic photon flux density was set as 500 μ mol m^−2^ s ^−1^, and CO_2_ was set at 400 μ mol mol^−1^. The chamber temperature was 20°C and the vapor pressure deficit was 0.5–1.0 kPa.

### Statistical analysis

The data, including PS VII, PS H, PS II, and PS I levels and sugar quantification results, were analyzed using one-way ANOVA (SPSS 19.0), and t tests were used to assess the significant differences between the 100- and 400-μmol m^−2^ s^−1^ treatments (*p ≤ 0.05*). Empirical polynomial (inverse third order) equations were used in Origin 2021 (Origin Pro Learning Edition) to analyze the relationship between PS VII and sucrose concentration in leaves (Zhou et al., 2020). Histograms representing chlorophyll concentration, fluorescence, and photosynthetic rate were generated using Origin 2021 (Origin Pro Learning Edition).

## Results

### PS VII has the highest concentration in leaves of medicinal *Paris* species

**Figure 4:**
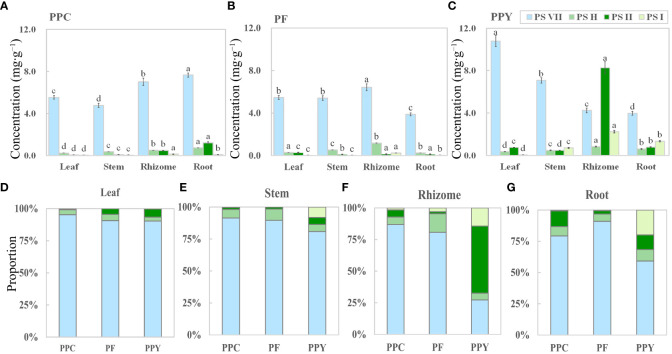
The organ-specific distribution of saponins in medicinal *Paris* species. **(A–C)** The concentration of PS VII, PS H, PS II, and PS I in four organs of PPC, PF, and PPY (mg g^−1^), respectively. **(D–G)** Proportion of PSs in the leaves, stems, rhizomes, and roots of PPC, PF, and PPY (%), respectively. PS, *Paris* saponins; PPC, *Paris polyphylla* var. *chinensis*; PF, *Paris fargesii*; PPY, *Paris polyphylla* var. *yunnanensis*. Each column represents the mean (± SE) of three replicates. The letters on the columns mean significantly different in the same saponin type between organs (*p ≤* 0.05).

PS VII, H, II, and I concentrations were analyzed in various organs, including leaves, stems, rhizomes, and roots (**Figure 4**). Four saponins were found in the leaves, stems, rhizomes, and roots of PPC, PF, and PPY. However, the concentrations (**Figures 4A–C**) and proportions (**Figures 4D–G**) of these saponins in the four organs varied among the different plant species. The main saponin in the leaves, stems, and roots of the three *Paris* species was PS VII (**Figures 4D, E, G**), especially in leaves and stem, where the proportion of PS VII was up to 80–90% (**Figures 4D, E**). In the rhizome, PS VII was also the main saponin in PPC and PF, but not in PPY (**Figure 4F**). In the rhizome of PPY, PS I had highest concentration, followed by PS VII. These findings suggest that PS VII is present in all organs of the three *Paris* species and its concentration is dominant compared with the other detected saponins. As a result, PS VII was chosen as the focus of further research in this study.

### PS VII flow from leaf to rhizome through vascular tissues

To investigate the potential movement of PSs out of the leaves, an experiment was conducted by covering the leaves with aluminum foil to block incoming light. The concentrations of PSs and soluble sugars, such as sucrose, glucose, and fructose, were monitored at specific time intervals: 0, 24, 48, and 72 h after the covering was applied. The concentrations of PS VII, H, and II in leaves increased from 0 h to 48 h, but then decreased sharply from 48 h to 72 h (**Figures 2B–D**). By contrast, the sucrose concentration in leaves showed a decreasing trend as the covering time increased. Between 0 h and 48 h, there was a sharp decline in sucrose levels (from 292.55 to 184.70 mg g^−1^), followed by a stable period from 48 h to 72 h (184.70 to 174.34 mg g^−1^) (**Figure 2F**). The rate of reduction in sucrose concentration in leaves was notably faster than that of saponins. These suggest that the transport of sucrose from the leaves (source) to the rhizomes (sink) was more rapid, resulting in an increase in the concentration of PSs in the leaves during the initial 0 to 48 h. Although the sucrose concentration in the leaves remained stable between 48 h and 72 h, there was a continued reduction in PS concentration in the leaves, leading to a sharp decrease. The trend in PS VII concentration showed a significant correlation with the sucrose concentration in the leaves (**Figure 2G**). However, no significant changes were observed in the concentrations of PS I, glucose, and fructose throughout the 72-h period.

The decreased concentration of PS in leaves does not automatically prove that saponins are being transported out of the leaves, as they might be being broken down or synthesized into other substances. Therefore, the stem wound fluid, leaves, and stems of PPY were obtained to analyze the similarity of PS (**Figure 3**). In stem wound fluid, the saponin type and proportions of the four saponins were almost the same as that of stem and leaves, and PS VII was the main saponin (**Figures 3B–D**). This proved that saponins in PPY can be transported to other organs via the fibrovascular tissue of leaves and stem.

### ^13^C-glucose feeding traces specificity in PS VII biosynthetic organs

The above results have proved that the leaves were an important organ for the synthesis of PS VII in PPY. However, from these results, we cannot conclude that the leaf is the major organ in the synthesis of PS VII, as the rhizome also contains high concentrations of PS VII. To further prove hypothesis 1, we conducted ^13^C-glucose labeling experiments to trace the synthesis sites and pathways of PS VII in PPY. PS VII concentrations in four organs of PPY in four groups (**Figure 1A**) were detected by high resolution liquid chromatography-mass spectrometry. The results showed that only the ratio of PS VII molecules (M+2) ^−^/M^−^ increased significantly in the leaves of group 1 and group 3 (**Figure 1B**). In group 1, PS VII marked with two ^13^C was synthesized in leaves of PPY after spraying with ^13^C-glucose. In group 3, ^13^C-glucose was feeding at the stem incision, and PS VII marked with two ^13^C could also be synthesized in leaves. However, the ratio of PS VII molecules (M+2)^−^/M^−^ in stems, rhizomes, and roots was obviated, with no differences among groups 1, 2, and 3. All the results suggested that leaves are the main synthesis organ of PS VII in PPY.

At the same time, the ratios of (M+1) ^−^/M^−^, (M+3) ^−^/M^−^, and (M+4) ^−^/M^−^, i.e., marked with one, three, or four ^13^Cs in each organ, were not significantly different between all the groups (**Figures S2A–C**). The results are consistent with the saponin biosynthetic pathway. Acetyl-coenzyme A can be produced from glycolysis. And PSs, which are isoprenoid compounds, are biosynthesized *de novo* from acetyl-coenzyme A via the mevalonate (MVA) pathway in the cytoplasm or the 2-C-methyl-D-erythritol 4-phosphate (MEP) pathway in the plastids (Vranová et al., 2013). After plants have been fed exogenous ^13^C-glucose, acetyl-coenzyme A with two ^13^Cs is produced from ^13^C-glucose glycolysis and is then used to biosynthesize PS VII with a maximum probability of two ^13^C. Therefore, there are higher levels of PS VII marked with two ^13^C than other molecules in the leaves.

To certify the success of root ^13^C-glucose feeding, further analysis of the ^13^C/^12^C isotope ratio in rhizomes was carried out. The ^13^C/^12^C isotope ratios of groups 2 and 3 were much higher than those of groups 1 and 4 (**Figure 1C**). The results showed that ^13^C-glucose had significantly entered the rhizomes through feeding in groups 2 and 3.

### TEM subcellular chemical localization of PSs in leaves

**Figure 5:**
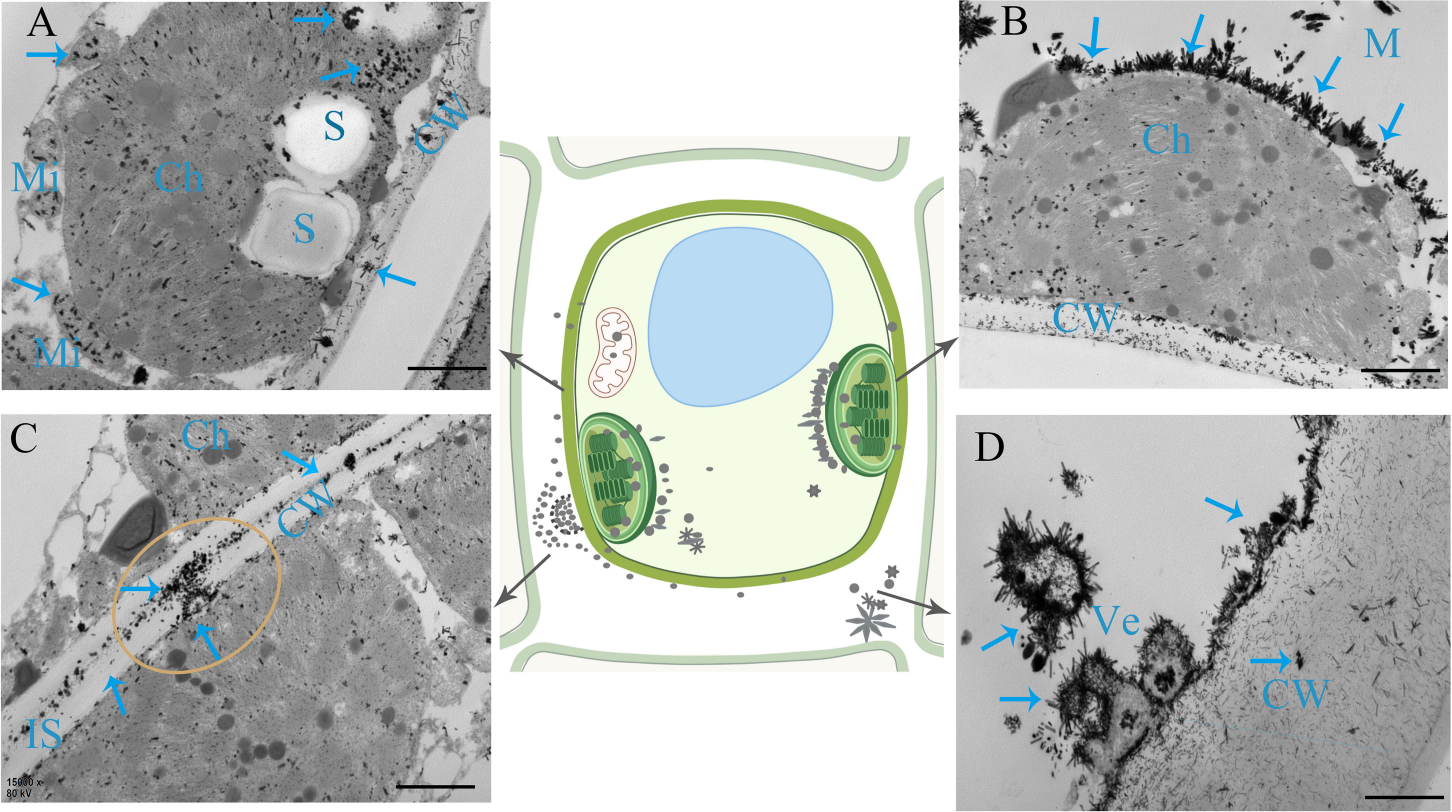
Subcellular chemical localization of PS in leaves. **(A)** Saponin precipitations can be observed as black masses positioned on the membrane. Chloroplasts (Ch), mitochondria (Mi), and cell walls (CW) are indicated by blue arrows. **(B)** Saponin precipitations in the form of needle crystals bundle positioned along the membrane. **(C)** Saponins are exocytosed into the intercellular space (IS) through transmembrane transport from the chloroplast and cell. **(D)** Saponin precipitations shed from the membrane into a small vesicle (Ve). S, starch grains. Bars=1 μm.

To visualize the specific sites of PS synthesis in leaves, subcellular chemical localization was carried out using TEM. The TEM images revealed the presence of saponin precipitations localized in various cellular components, including the membrane, chloroplasts, mitochondria, and cell wall (**Figure 5A**). These saponin precipitations appeared as needle crystals bundled along the membrane (**Figure 5B**). The exocytosis of saponins occurred from both the chloroplast and cell, leading to their release into the intercellular space via transmembrane transport. Subsequently, the saponins dispersed peripherally along the intercellular space, exhibiting a gradual reduction in abundance (**Figure 5C**). Additionally, the observations revealed instances in which saponin precipitations detached from the cellular membrane and entered small vesicles within the intercellular space or inside the cells (**Figure 5D**).

### Physiological response of PPY leaves under light intensity treatments

Based on the aforementioned findings, it is evidenced that the synthesis of PS VII primarily occurs in the leaves rather than the rhizome. To investigate the potential regulation of PS synthesis in leaves by light intensity, we initiated an examination of the photosynthetic and fluorescence properties of the leaves under two different light intensity treatments, 100 μmol m^-2^ s^-1^ and 400 μmol m^-2^ s^-1^. This initial assessment was conducted to ensure the appropriateness of the selected light intensity treatments for further investigations.

**Figure 6:**
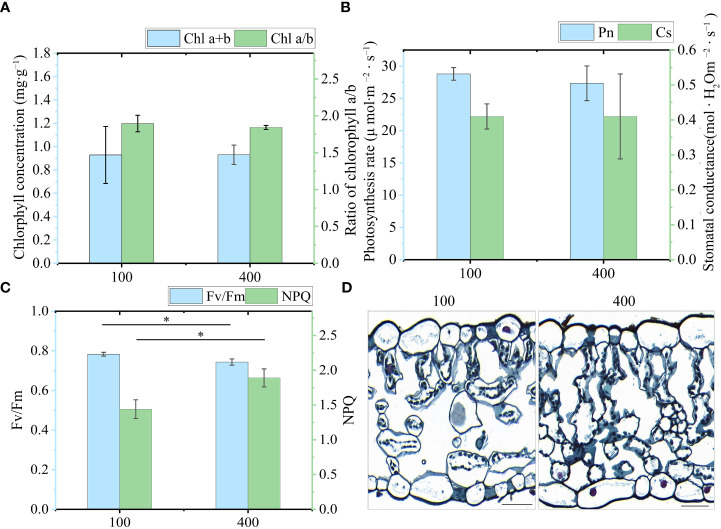
Physiological response of leaves under two light intensities. **(A)** Chlorophyll a+b (Chl a+b) and Chlorophyll a/b (Chl a/b). **(B)** Photosynthesis rate (Pn) and stomatal conductance (Cs). **(C)** *Fv/Fm* and NPQ (Fm/Fm’-1). **(D)** Characteristics of a transverse leaf section under 100 μmol m^−2^ s^−1^ and 400 μmol m^−2^ s^−1^ light intensity. Bar=50 μm. Each column represents the mean (± SE) of three replicates. * indicates a significant difference between the given light treatments (t-test at *p ≤ 0.01*).

Total chlorophyll (Chl a+b), chlorophyll a/b, photosynthetic rate, and Cs showed no difference between the two light intensity treatments (**Figures 6A, B**). However, chlorophyll fluorescence identified a slight inhibition of the photosynthetic system under 400 μmol m^−2^ s^−1^ (**Figure 6C**). The level of *Fv/Fm* under 400 μmol m^−2^ s^−1^ was lower than that of leaves under 100 μmol m^−2^ s^−1^. By contrast, the NPQ of leaves under 400 μmol m^−2^ s^−1^ was significantly higher than that under 100 μmol m^−2^ s^−1^. In addition, we examined the anatomical characteristics of the leaves to further visualize the effect of light intensity on leaf morphology (**Figure 6D**). Plants exhibit adaptive responses to different light intensities through morphological changes, including alterations in cell morphology and chloroplast arrangement. Under 100 μmol m^-2^ s^-1^, there was a reduced number of mesophyll cells, with chloroplasts arranged laterally along the cells. This arrangement provided a larger chloroplast surface area exposed to light, facilitating photosynthesis. By contrast, leaves subjected to 400 μmol m^-2^ s^-1^ exhibited an increased number of mesophyll cells, and chloroplasts were arranged more closely together longitudinally. Taken together, these results indicate that 400 μmol m^−2^ s^−1^ in the greenhouse should be an appropriate light intensity for investigating whether the synthesis of PSs in leaves is regulated by high light intensity, as this had a slight photoinhibitory effect on PPY leaves but did not affect their photosynthetic capacity.

### PS VII concentration in different organs and tissues of leaves in response to light intensities

**Figure 7:**
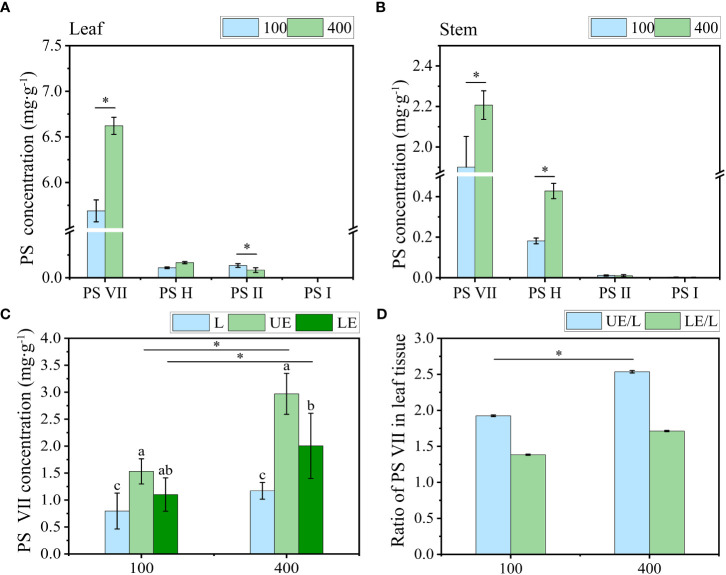
The effect of light intensity on PS concentration in **(A)** leaf and **(B)** stem. **(C)** Concentration of PS VII in leaf tissue without vein (L), upper epidermal cells (UE) and lower epidermal cells (LE). **(D)** Ratio of PS VII in leaf tissue of PPY. PS, *Paris* saponins. Each column represents the mean (± SE) of three replicates. * indicates s significant difference between the two given light intensity treatments (t-test at *p ≤ 0.01*). Data with different letters are significantly different between the partial leaf tissue (*p ≤ 0.05*).

The concentration of PS VII in leaves under 400 μmol m^−2^ s^−1^ was significantly higher than that under 100 μmol m^−2^ s^−1^ (**Figure 7A**). Similarly, in the stem, the concentration of PS VII was also higher under 400 μmol m^−2^ s^−1^ than under 100 μmol m^−2^ s^−1^ (**Figure 7B**). However, no significant difference in PS VII concentration was observed between the two light intensity treatments in the rhizome and root (**Figure S3**).

To further understand how high light intensity increases PS synthesis, we quantified the level of PS VII in tissues (including leaf tissue without vein, upper epidermal cells, and lower epidermal cells) of the leaf under two light intensity treatments. Under both light intensity treatments, the level of PS VII in the upper epidermal cells, low epidermal cells, and leaf tissue sequentially declined (**Figure 7C**). High light intensity (400 μmol m^−2^ s^−1^) increased PS VII concentration in all three leaf tissues, particularly the upper epidermal cells. The ratio of PS VII concentration in the upper epidermal cells to that in the entire leaf tissue was 2.5 under 400 μmol m^−2^ s^−1^, which was 31.6% higher than the ratio observed under 100 μmol m^−2^ s^−1^ (**Figure 7D**).

**Figure 8:**
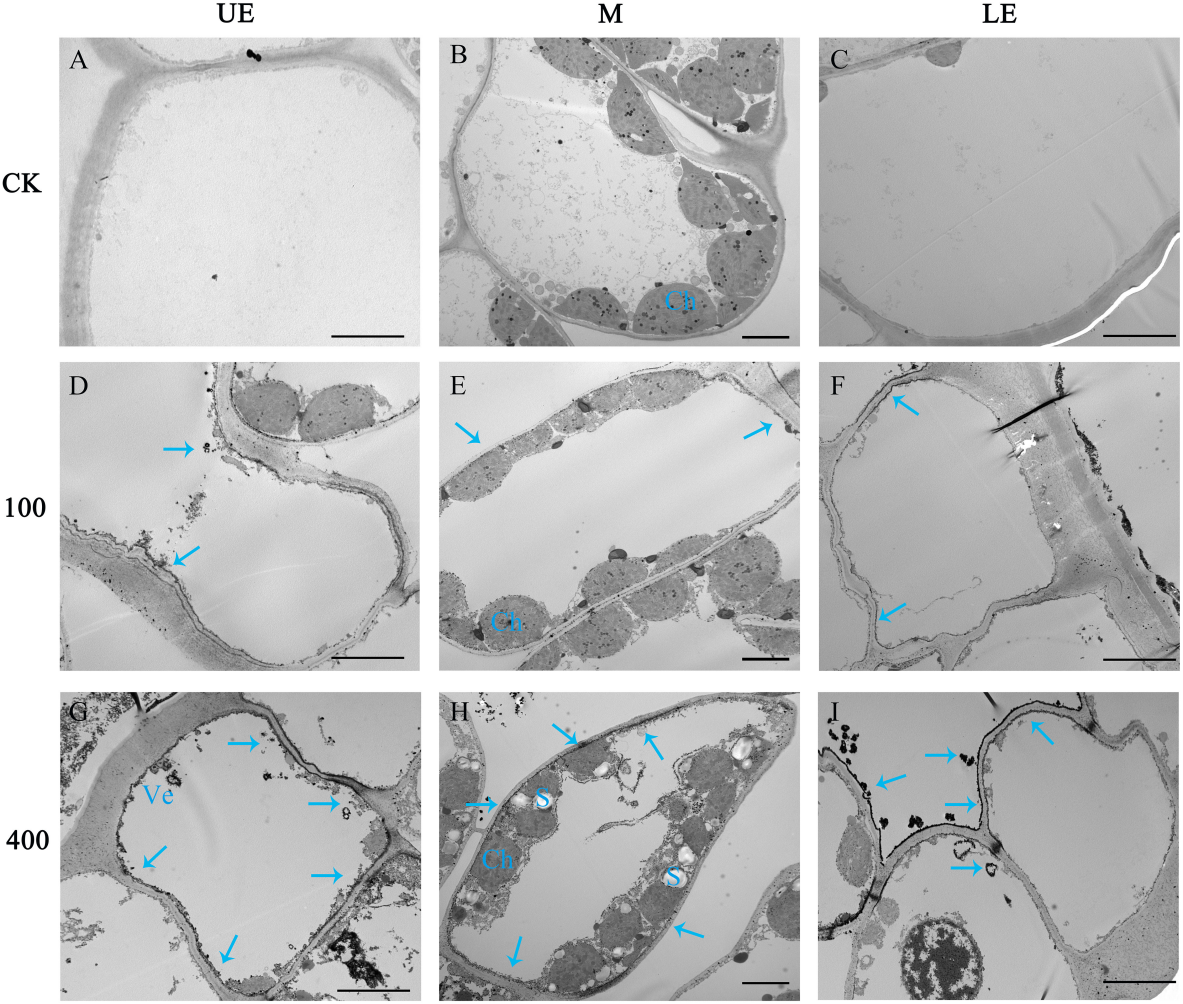
The effect of light intensity on the distribution of saponins in leaf cells. Saponin precipitations can be observed as black masses indicated with blue arrows. **(A–C)** Group CK with no saponin precipitation. **(D–F)** PPY leaves under LED 100 μmol m^−2^ s ^−1^. **(G–I)** PPY leaves under LED 400 μmol m^−2^ s ^−1^. UE, upper epidermal cell; M, mesophyll cell; LE, lower epidermal cell; Ch, chloroplast (Ch); Ve, vesicle. Bars=5 μm **(B, E, H)**; 10 μm **(A, C, D, F, G, I)**.

The differential distribution of PSs in leaf tissue under the two light intensity treatments was further confirmed through the cytochemical localization of saponins using TEM (**Figure 8**). In comparison to the 100 μmol m^−2^ s^−1^ treatment (**Figures 8D–F**), a greater number of saponin precipitations were observed in the upper and lower epidermis leaves exposed to a light intensity of 400 μmol m^−2^ s^−1^ (**Figures 8G–I**). Conversely, the control group (CK) showed no saponin precipitations (**Figures 8A–C**). These findings suggest that the increase in PS synthesis in leaves under high light intensity is accompanied by enhanced translocation and accumulation of saponins in the upper epidermal cells. This phenomenon may contribute to improved resistance against photodamage.

## Discussion

The purpose of this study was to investigate the role of the leaf in PS VII synthesis and to explore the influence of light intensity on this process.

### The leaf is a major organ in the synthesis of PS VII

Studies on various plant species have shown that the synthesis and accumulation of saponins can occur in different organs, emphasizing the important role of leaves in saponin synthesis. For example, studies on *Achyranthes bidentata*, *Polygala tenuifolia*, and *Cyclocarya paliurus* have found high saponin concentrations in leaves, particularly in the palisade tissue (Li and Hu, 2009; Teng et al., 2009; Chen et al., 2019). The presence of saponins in the stem vascular bundles of plants such as *Achyranthes bidentata*, *Panax notoginseng*, and *Panax ginseng* suggests that stems can also act as transport organs for saponins (Li and Hu, 2009; Yokota et al., 2011; Mu et al., 2016). In the case of *Dioscorea zingiberensis*, comprehensive research has revealed that diosgenin saponins are synthesized in the leaves and then transported through the stem to the rhizome for storage, supported by the specific expression of diosgenin biosynthetic genes in the leaves (Li et al., 2022). These findings align with the observation that the accumulation of rhizome saponins in perennial plants gradually increases with age during the initial years (Wang and Li, 2018). Additionally, in the present study, we observed high concentrations of saponins, particularly PS VII, in the leaves of PPC, PF, and PPY, and identified the transport of saponins from leaves to the rhizome through the stem (**Figure 3**). However, it is important to note that these results alone do not conclusively establish that leaves are the main organ for the synthesis of PS VII.

Although the genome of PPY has been sequenced, the specific genes and enzymes responsible for regulating the synthesis of PS VII remain unknown (Li et al., 2020). In light of this, rather than adopting genetic approaches, we opted for physiological methods to explore the specific organ responsible for PS VII synthesis. We used ^13^C labeling in our study, which is a well-established method for tracing the synthesis sites and pathways of substances (Wang et al., 2018; Wu et al., 2022). To expedite the experiment and ensure the reliability of our results, the plants were fed with ^13^C-glucose, which is rapidly metabolized to acetyl-CoA, a precursor for saponin synthesis (Vranová et al., 2013). In both the leaf and stem vascular bundle feeding treatments, the ratio of ^13^C-labeled PS VII to unlabeled PS VII in the leaves was higher than the ratio in the control group (**Figure 1B**). Conversely, in the rhizome and stem vascular bundle feeding treatments, there was no significant difference in the ratio of ^13^C-labeled PS VII to unlabeled PS VII in the rhizome between the labeling and non-labeling treatments (**Figure 1B**). The concentration of ^13^C in the rhizome was quantified and found to be significantly higher in the two labeling treatments than in the no feeding treatment (**Figure 1C**), confirming the successful labeling of the rhizome with ^13^C-glucose. Additionally, the process of saponin release from the cells into the intercellular space in the leaves, followed by diffusion along the intercellular space, was observed (**Figure 5C**). Interestingly, PS VII labeled with ^13^C was not detected in the rhizome when the leaves were fed with ^13^C-glucose, which challenges the notion that PS VII is transported from the leaves through the stem to the rhizome. One possible explanation for this discrepancy is that the transit speed of saponins from leaves to rhizome is slow, as indicated by the lack of decrease in PS VII concentration after the leaves were covered with foil for 48 h (**Figure 2B**).

Although the synthesis of saponins is typically attributed to the leaves in the majority of reported plant species, it cannot be regarded as an absolute rule. In our current study, intriguingly, the rhizome of PPY exhibited the highest proportion of PS II, albeit at a significantly lower concentration than PS VII in the leaves (**Figure 4C**). Additionally, there was no difference in the ratios of ^13^C-labeled PS II to unlabeled PS II in the leaves of plants under leaf-feeding and non-feeding treatments (**Figures S2D–G**), indicating that the leaves may not be the main site of PS II synthesis in PPY. This conclusion may contradict previous findings reported in the literature. One possible explanation might be that most of the studies conducted focused only on total saponin levels and tissue localization, without detailing the saponin types, ignoring the tissue specificity of different types of saponin synthesis. Therefore, the question is why the leaves specifically synthesize PS VII. The reason for this can mainly be attributed to the chemical structural and functional properties of PS VII, whereby: (1) unlike most of the saponins in PPY, including PS H, I, dioscin, and gracillin, which are linked with a trisaccharide chain (Li, 1998), PS VII has a tetrasaccharide chain linked at the C-3 position, providing greater resistance to biotic and abiotic stresses in field conditions, including predation, pathogenic microorganisms, and high light intensity. Additionally, pharmacological studies have shown that PS VII exhibits stronger cytotoxic and anti-inflammatory properties than saponins with a trisaccharide chain (Fan et al., 2015; Zhang et al., 2019; Ahmad et al., 2021); (2) the synthesis of PS VII in leaves aligns with the concept of phyto-economics, as it is more convenient and requires less energy (Züst and Agrawal, 2017; He et al., 2022). Owing to the glycosidic linkage of PS VII, it can be enzymatically modified, leading to the formation of other saponins through the relinking of sugar chains or changes in the linkage position (Hua et al., 2022; Song et al., 2022). However, we still lack more direct and convincing evidence of the variability of the synthetic organization of the saponin types in *Paris* species and other plant species with saponins, and further work is needed to unravel their mystery so that a contribution can be made to the production of high-quality *Paris* herbs and human health. Nevertheless, these results certify that the leaf of PPY is a major organ in the synthesis of PS VII.

### PS VII synthesis in leaves regulated by light intensity

The synthesis, transport, and distribution of plant metabolites play a crucial role in plant defense against biotic stresses. For instance, plants protect themselves from attackers by using specialized metabolites and from herbivores by optimizing the distribution of chemical defenses (Hunziker et al., 2021). Mature leaves often allocate defense compounds to vulnerable or reproductive parts of the plant to enhance defense mechanisms against herbivory (Hunziker et al., 2021; Gershenzon and Ullah, 2022). Additionally, saponins in plants such as *Centella asiatica* have been found to increase resistance against UV oxidation damage (Müller et al., 2013). Additionally, the level of leaf saponin concentration can influence the response to high light stress, with variations observed between young and old leaves (Chen et al., 2019). Nevertheless, the specific mechanisms underlying saponin synthesis, distribution, and accumulation in leaves in response to abiotic stresses, particularly light intensity, are poorly understood.

The present study revealed that plants grown under a light intensity of 400 μmol m^−2^ s^−1^, which slightly inhibited the growth of PPY in the controlled environment, exhibited higher PS VII concentration in leaves than those grown under 100 μmol m^−2^ s^−1^. Additionally, the distribution of saponins in leaf cell types was also regulated, with a higher proportion of saponins found in the leaf epidermal cells of plants grown under 400 μmol m^−2^ s^−1^. The explanations for the light-induced regulation of saponin synthesis and distribution may be based on the following aspects: (1) high light intensity increases the availability of substrates for saponin synthesis, thus driving the reaction in a positive direction. This is supported by the higher number of starch granules observed under 400 μmol m^−2^ s^−1^ than under 100 μmol m^−2^ s^−1^ (**Figure 8H**). (2) Similar to the role of saponins in resistance to UV oxidative damage (Müller et al., 2013), the increased synthesis and distribution of saponins in epidermal cells under high light intensity may help protect against high light damage. (3) High light intensity promotes the transport of saponins to epidermal cells, and this feedback regulation may further modulate saponin synthesis. Compartmentalization of secondary metabolism, such as saponin synthesis, can effectively control the rate and direction of metabolic flow, reduce the cytotoxicity of intermediates, and minimize the metabolic burden on cells (Sun et al., 2023). The slow rate of PS VII translocation between organs (**Figure 2**), suggests that compartmentalization of PS VII in leaves is an effective strategy for maintaining its synthesis rate. However, how light intensity regulates saponin synthesis and distribution in leaves is still unclear, and further work is needed to elucidate the underlying mechanisms.

Indeed, understanding the relationship between light and the expression and signaling pathways of key genes involved in saponin synthesis, such as *PgMYB2*, *PgMYC2*, *PgWRKY1*, and *PgWRKY22*, and genes including *PgDELLA*, *PnDS*, and *PnSE* (Rahimi et al., 2016; Deng et al., 2017; Liu et al., 2019b; Zheng et al., 2023), is crucial in unraveling the light-mediated regulation of saponin synthesis in *Paris* plants. These transcription factors and genes have been shown to play important roles in the light-mediated regulation of saponin synthesis in *Panax*. Further investigation into the expression patterns and signaling pathways of these genes in *Paris* plants during saponin synthesis will provide valuable insights into the molecular mechanisms underlying the light regulation of saponin metabolism. By gaining a higher-order understanding of these regulatory processes, it will be possible to increase the production of high-quality medicinal materials from *Paris* plants through environmentally friendly and resource-efficient cultivation practices.

## Conclusion

In the present study, chemical phenotypic analysis, subcellular localization, and ^13^C tracing have demonstrated at the physiological level that the leaf rather than the rhizome of PPY is the main organ for the synthesis of PS VII. However, PS VII is not stored in the leaves but is instead transported through the vascular tissue to the rhizome for storage. The synthesis of PS VII in leaves is regulated not only by genetics but also by environmental cues, such as light intensity. The proposed physiological mechanisms underlying the positive effects of high light intensity on PS VII synthesis are as follows: (1) high light intensity increases the availability of substrates required for saponin synthesis; (2) promoting saponin synthesis protects leaves from potential damage caused by high light; and (3) high light intensity enhances the compartmentalization of saponins within the leaves, including transfer to the upper epidermal cells, which in turn feedback regulates saponin synthesis. These studies partially unveil the tissue-specific and light-regulated properties of PS VII synthesis in PPY and lays the theoretical foundation for the development of double high (high yield and high quality) cultivation practices. The tissue specificity of the synthesis of saponin types and the genetic and ecological mechanisms of light-regulated saponin synthesis should be the focus of future studies.

## Data availability statement

The original contributions presented in the study are included in the article/**Supplementary Material**. Further inquiries can be directed to the corresponding authors.

## Author contributions

FW, TZ, and JP planned and designed the study and wrote the manuscript. FW, SC, and YW cultivated the plants and carried out the chemical analysis experiments and statistical analysis. QW and JY conducted the fieldwork. FW and TZ analyzed the data. FW and SC contributed equally. All authors contributed to the article and approved the submitted version.
