# Supplementary material for: The synthesis of Paris saponin VII mainly occurs in leaves and is promoted by light intensity
Source: Front Plant Sci. 2023 Jul 28;14:1199215. doi: 10.3389/fpls.2023.1199215 (PMC10420111; doi:10.3389/fpls.2023.1199215)
Supplement: Supplementary file 1 [file DataSheet_1.docx]

Fig. S1


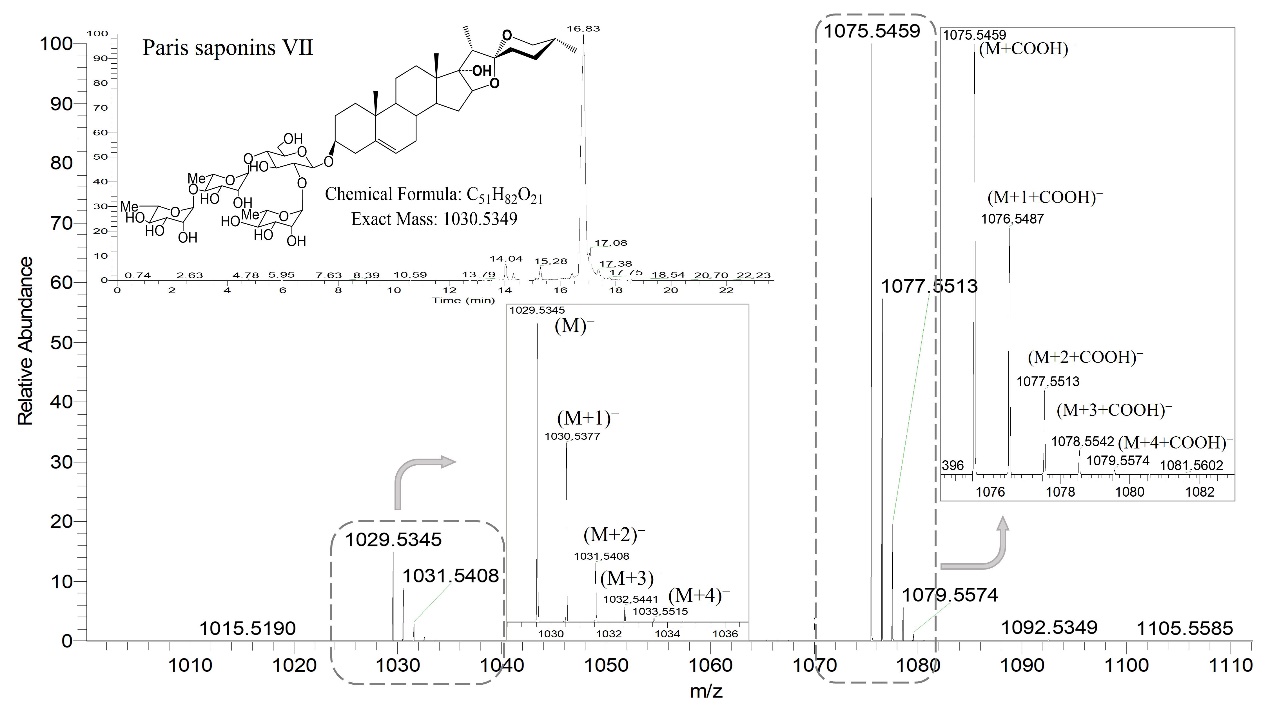


**Figure S1. PS Ⅶ ion bar diagram by UPLC-mass spectrometry.**

Negative ion by high-resolution mass spectrometry of PS Ⅶ (C_51_H_82_O_21_, 1030.5349) was detected with a retention time of 16.83 min. Owing to the natural abundance of ^13^C with the exact molecular weight of 13.003 (^12^C 12.000), there will be a series of isotope peaks exiting in the same retention time position of the PS Ⅶ molecular ion peak (M^−^), mainly (M+1)^−^, (M+2)^−^, (M+3)^−^ and (M+4)^−^, etc. The detailed figure displays an enlarged bar diagram of the series isotope peaks. The calculation of the ion current ratio is (M+1)^−^/M^−^, (M+2)^−^/M^−^, (M+3)^−^/M^−^, and (M+4)^−^/M^−^. The ratio changes of the series isotope ion peaks were used to track and infer the biosynthesis of saponins. M^−^ means the sum of the peak areas of ion current (M+COOH)^−^ and M^−^, which were mainly ionized with COOH^−^ ions in formic acid water.

Fig. S2


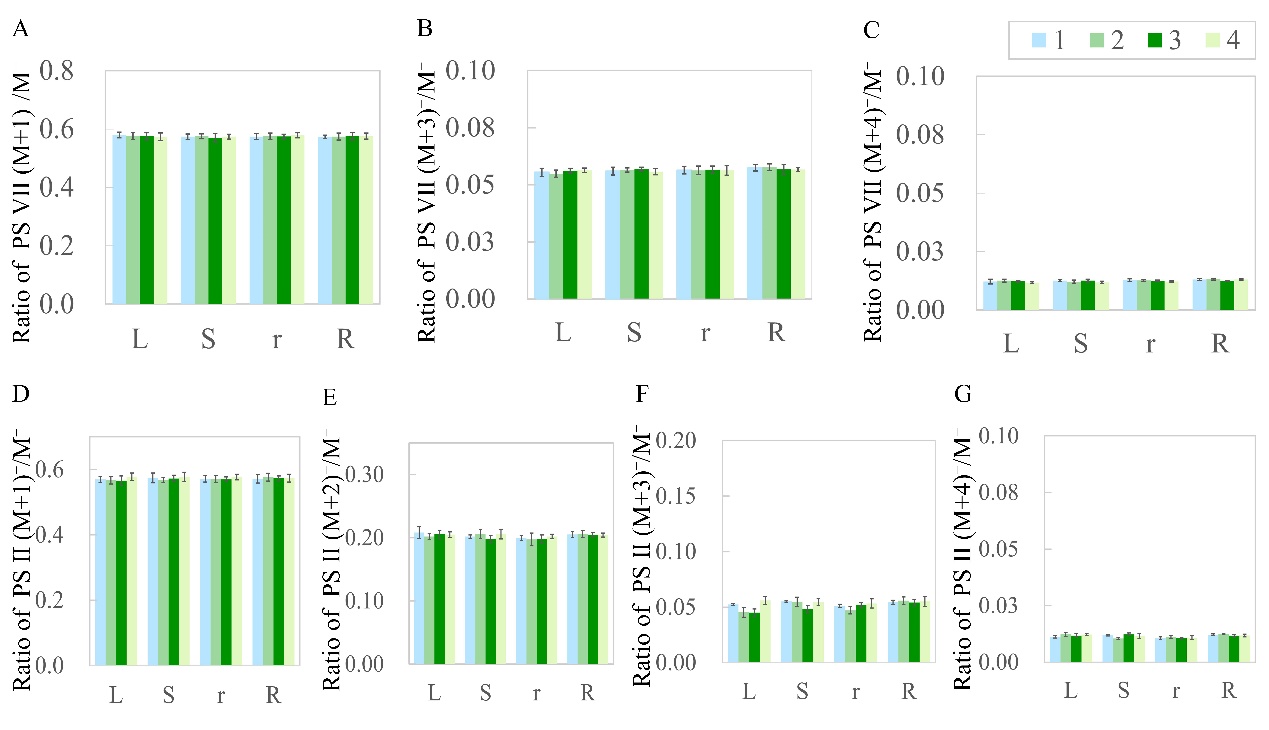


**Figure S2. The ratios of PS Ⅶ and PS Ⅱ in organs of the four groups via ^13^C-glucose (^13^C_6_H_12_O_6_) feeding.**

(A–C) The ratios of PS Ⅶ (M+1)^−^/ M^−^, (M+3)^−^/ M^−^, and (M+4)^−^/ M^−^ in each organ of the four groups were not significantly different. (D–G) The ratios of PS Ⅱ (M+1)^−^/ M^−^, (M+2)^−^/ M^−^, (M+3)^−^/ M^−^, and (M+4)^−^/ M^−^ in each organ of the four groups were not significantly different. L, leaf; S, stem; r, root; R, rhizome. Each column represents the mean (± SE) of three replicates.

Fig. S3


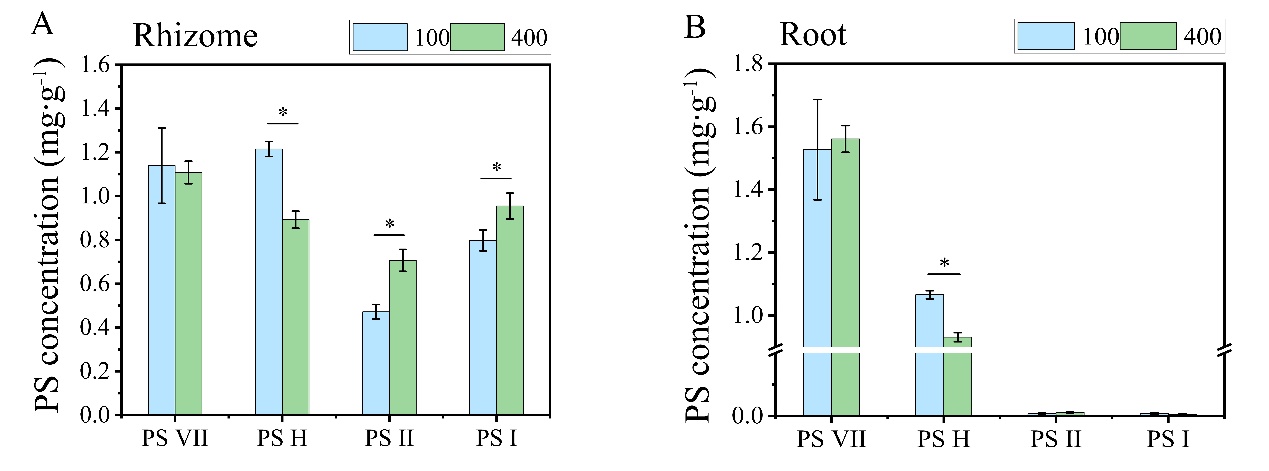


**Figure S3.** **Concentration of saponins in the root and rhizome of PPY under different light intensities.**

The saponins of 2-year-old PPY seedlings under light intensities of 100 and 400 μmol m^−2^ s^−1^ for 1 week. PS H in the root and rhizome of 100 μmol m^−2^ s^−1^ are a little higher than that of 400 μmol m^−2^ s^−1^. PS Ⅱ and PS I in the root and rhizome of 400 μmol m^−2^ s^−1^ are a little higher than that of 100 μmol m^−2^ s^−1^. PS Ⅶ, PS H, PS Ⅱ, PS Ⅰ, *Paris* saponin Ⅶ, H, Ⅱ, and Ⅰ, respectively. * indicates a significant difference between the same saponin components at a given light treatment (t-test at *p ≤ 0.01*).
